# Composition of the sputum bacterial microbiome of patients with different pathomorphological forms of non-small-cell lung cancer

**DOI:** 10.18699/vjgb-24-25

**Published:** 2024-04

**Authors:** V.G. Druzhinin, E.D. Baranova, P.S. Demenkov, L.V. Matskova, A.V. Larionov

**Affiliations:** Kemerovo State University, Kemerovo, Russia Kemerovo State Medical University, Kemerovo, Russia; Kemerovo State University, Kemerovo, Russia; Institute of Cytology and Genetics of the Siberian Branch of the Russian Academy of Sciences, Novosibirsk, Russia; Karolinska Institute, Stockholm, Sweden; Kemerovo State University, Kemerovo, Russia

**Keywords:** non-small cell lung cancer, squamous cell lung cancer, lung adenocarcinoma, bacterial microbiome, sputum, taxonomic composition, 16S rRNA, NGS sequencing, немелкоклеточный рак легкого, плоскоклеточный рак легкого, аденокарцинома легкого, бактериальный микробиом, мокрота, таксономический состав, 16S рРНК, NGS секвенирование

## Abstract

Recent studies have shown that the bacterial microbiome of the respiratory tract influences the development of lung cancer. Changes in the composition of the microbiome are observed in patients with chronic inflammatory processes. Such microbiome changes may include the occurrence of bacteria that cause oxidative stress and that are capable of causing genome damage in the cells of the host organism directly and indirectly. To date, the composition of the respiratory microbiome in patients with various histological variants of lung cancer has not been studied. In the present study, we determined the taxonomic composition of the sputum microbiome of 52 patients with squamous cell carcinoma of the lung, 52 patients with lung adenocarcinoma and 52 healthy control donors, using next-generation sequencing (NGS) on the V3-V4 region of the bacterial gene encoding 16S rRNA. The sputum microbiomes of patients with different histological types of lung cancer and controls did not show significant differences in terms of the species richness index (Shannon); however, the patients differed from the controls in terms of evenness index (Pielou). The structures of bacterial communities (beta diversity) in the adenocarcinoma and squamous cell carcinoma groups were also similar; however, when analyzed according to the matrix constructed by the Bray–Curtis method, there were differences between patients with squamous cell carcinoma and healthy subjects, but not between those with adenocarcinoma and controls. Using the LEFse method it was possible to identify an increase in the content of Bacillota (Streptococcus and Bacillus) and Actinomycetota (Rothia) in the sputum of patients with squamous cell carcinoma when compared with samples from patients with adenocarcinoma. There were no differences in the content of bacteria between the samples of patients with adenocarcinoma and the control ones. The content of representatives of the genera Streptococcus, Bacillus, Peptostreptococcus (phylum Bacillota), Prevotella, Macellibacteroides (phylum Bacteroidota), Rothia (phylum Actinomycetota) and Actinobacillus (phylum Pseudomonadota) was increased in the microbiome of sputum samples from patients with squamous cell carcinoma, compared with the control. Thus, the sputum bacterial microbiome of patients with different histological types of non-small-cell lung cancer has significant differences. Further research should be devoted to the search for microbiome biomarkers of lung cancer at the level of bacterial species using whole-genome sequencing.

## Abstract (ru)

Исследования последних лет показали, что бактериальный микробиом респираторного тракта влияет на развитие рака легкого. Изменение состава микробиома у пациентов связывают с хроническими воспалительными процессами, так как многие бактерии вызывают окислительный стресс, а также способны прямо или опосредованно повреждать геном в клетках организма хозяина. До настоящего времени состав респираторного микробиома у больных с различными гистологическими вариантами рака легкого не изучен. В настоящем исследовании для анализа таксономического состава микробиома мокроты 52 пациентов с плоскоклеточным раком легкого, 52 пациентов с аденокарциномой легкого и 52 здоровых доноров контрольной группы использовали технологию массового параллельного секвенирования региона V3-V4 16S рРНК. Микробиомы мокроты больных с разными гистологическими типами рака легкого и контроля не имели значимых различий по индексу видового богатства (Шеннона), однако у пациентов они отличались от контроля по индексу выравненности (Пиелу). Структуры бактериальных сообществ (бета-разнообразие) между аденокарциномой и плоскоклеточным раком также были близкими. Тем не менее матрица, построенная по Брэю–Кёртису, позволила выявить различия между пациентами с плоскоклеточным раком и здоровыми субъектами, но не между аденокарциномой и контролем. Метод LEFse позволил идентифицировать в мокроте больных плоскоклеточным раком увеличение содержания Bacillota (Streptococcus и Bacillus) и Actinomycetota (Rothia) при сопоставлении с образцами пациентов с аденокарциномой. Не найдено различий в содержании бактерий между образцами больных аденокарциномой и контроля. В микробиоме образцов мокроты пациентов с плоскоклеточным раком по сравнению с контролем было повышено содержание представителей родов Streptococcus, Bacillus, Peptostreptococcus (филум Bacillota), Prevotella, Macellibacteroides (филум Bacteroidota), Rothia (филум Actinomycetota) и Actinobacillus (филум Pseudomonadota). Таким образом, бактериальный микробиом мокроты пациентов с разными гистологическими типами немелкоклеточного рака легкого имеет существенные различия. Дальнейшие исследования должны быть посвящены поиску микробиомных биомаркеров рака легкого на уровне бактериальных видов с использованием полногеномного секвенирования.

## Introduction

Recent studies show that many bacteria living in the human body are related to the development of malignant tumors. Microbial ecosystems capable of initiating oncogenic transformation, inducing metabolic changes in the tumor microenvironment, or modulating responses to cancer immunotherapy have already been described (Xavier et al., 2020; Chen et al., 2022). Integrated metagenomic approaches are expected to accurately identify tumor-associated microbiome profiles and uncover mechanisms of bacterial influence on cancer initiation and progression (Chiu, Miller, 2019). Moreover, recent studies have identified microbial profiles specific to certain cancer types that may serve as biomarkers for diagnosing tumor risk (Wu et al., 2021).

Lung cancer (LC) originates in the lung parenchyma or bronchi and is diagnosed in approximately 1.2 million people worldwide each year (Cheng T.Y. et al., 2016). Mortality from LC remains high, in part due to the lack of early detection of diagnostic biomarkers, including metagenomic markers. Therefore, the search for bacteria associated with the risk of developing LC has intensified dramatically in recent years, especially with the application of massively parallel DNA sequencing technology (Mao et al., 2018; Maddi et al., 2019). Previous studies have shown that there are features of microbiota composition in saliva, bronchoalveolar lavage, and lung tissue samples that may be associated with LC, but the results of these studies regarding the significance of specific bacteria are largely contradictory (Hasegawa et al., 2014; Lee et al., 2016; Liu H.X. et al., 2018; Tsay et al., 2018; Peters et al., 2019; Wang et al., 2019; Zhang et al., 2019; Cheng C. et al., 2020; Zhuo et al., 2020).

An important source of information on the composition of the respiratory tract microbiota is sputum, which has so far been little studied in LC patients (Hosgood et al., 2014, 2019; Cameron et al., 2017; Druzhinin et al., 2020; Ran et al., 2020). Although sputum does not reflect the microbiome of any specific part of the respiratory tract, it may be useful for searching for metagenomic biomarkers of LC because its collection is relatively simple and non-invasive.

Despite the fact that all forms of LC originate from epithelial cells of the airway mucosa, the current classification includes several different histologic types of this disease (Tsao, Yoon, 2018). LC is commonly divided into small cell lung cancer and non-small cell lung cancer (NSCLC), which accounts for 85 % of all LC cases (Molina et al., 2008). NSCLC is in turn subdivided into large cell lung cancer, adenocarcinoma of the lung (AD), and squamous cell lung cancer (LUSC). Different histological types of LC are characterized by distinctive biological patterns, different molecular markers and specific treatment strategies (Herbst et al., 2008). Based on this, it can be hypothesized that the composition of the respiratory tract microbiome may also differ between AD and LUSC patients. To date, this question remains open, given the very few published studies comparing the respiratory microbiome with individual histologic types of LC.

Here, we present the results of a comparative study of the taxonomic composition of the bacterial microbiome of the sputum of AD, LUSC patients and healthy donors, residents of the Kuzbass region of Western Siberia, for the first time.

## Materials and methods

Microbiota composition was studied in sputum samples from 52 patients with AD (37 men, 15 women; mean age 62.5 years); 52 patients with LUSC (49 men, 3 women; mean age 59.9 years) and 52 healthy donors (39 men, 13 women; mean age 62.5 years). The cohort of patients with NSCLC was formed from individuals who were first admitted for examination to the Kemerovo Regional Oncology Center (Kemerovo, Russian Federation). The material for the study was collected from March 2018 to March 2022. A questionnaire was filled out for each participant with information on place and date of birth, living environment, occupation, exposure to occupational hazards, health status, medication intake, radiologic procedures, smoking and alcohol consumption. For patients with NSCLC, the results of clinical and histological analyses, primary tumor localization, and disease stage according to the TNM classification were additionally taken into account (Goldstraw, 2013). Demographic and clinical data on patients and control donors are presented in Table 1

**Table 1.**
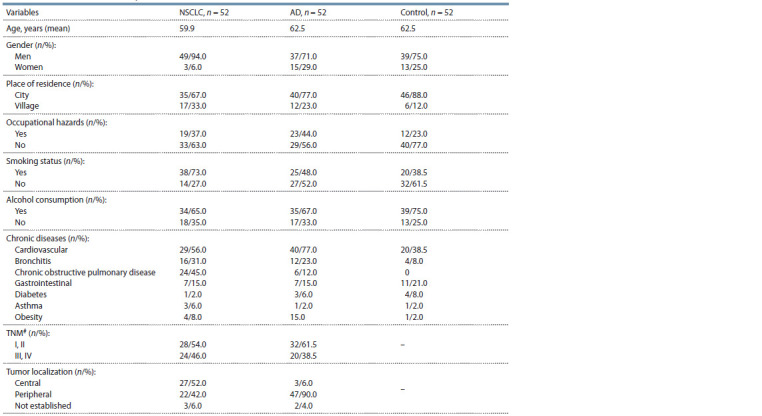
Characteristics of the study cohorts # TNM – tumor-node-metastasis

Inclusion criteria were male and female age ≥ 40 years, sputum donation, and signing written informed consent. Exclusion criteria were any acute or chronic condition that would limit the patient’s ability to participate in the study, use of antibiotics within 4 weeks prior to collection, inability to obtain a sputum sample, or refusal to give informed consent. All participants were informed about the aims, possible risks of the study and signed informed consent. The study was approved by the Biomedical Ethics Commission of Kemerovo State University (protocol # 17/2021 dated 05.04.2021). When patients and control donors were included in the study, ethical principles required by the World Medical Association Declaration of Helsinki (World Medical Association Declaration of Helsinki, 1964, 2000) were followed.

To analyze the taxonomic composition of the respiratory microbiome, sputum samples (2–3 ml) from patients with NSCLC and control group donors were obtained noninvasively through productive coughing. The obtained samples were immediately placed in sterile plastic vials and frozen (–20 °C). Frozen samples were transported to the laboratory and stored at –80 °C until bacterial DNA extraction.

DNA extraction, amplification, and sequencing of 16S rRNA on a MiSeq instrument (Illumina, USA) were performed according to the manufacturer’s recommendations. A detailed description of the procedures is given in a previous publication (Druzhinin et al., 2021).

Microbiome sequencing data were processed using the QIIME2 software package (Bolyen et al., 2019). Quality assurance was performed and a sequence library was created. Sequences were combined into operational taxonomic units (OTUs) based on a 99 % nucleotide similarity threshold using the Greengenes (version 13-8) and SILVA (version 138) reference sequence libraries, followed by removal of singletons (OTUs containing only one sequence). The correspondence of bacterial phylum names to current international nomenclature was determined using the LPSN resource (Parte et al., 2020).

The total diversity (alpha-diversity) of sputum prokaryotic communities was estimated by the number of isolated OTUs (analogous to species richness) and Shannon indices (H = Σpi ln pi, where pi is the proportion of the i-th species in the community). The evenness of species distribution in terms of their abundance in the community was assessed by the Pielou index. The difference in the structure of bacterial communities of different samples (beta diversity) was analyzed using UniFrac (Lozupone, Knight, 2005), a method common in microbial ecology that assesses the difference between communities based on the phylogenetic relatedness of the represented taxa. Normalization of samples by 1070 sequences (minimum number of sequences obtained per sample) was used to calculate diversity indices. The significance of differences between groups of samples was assessed by the PERMANOVA method (Adonis). The construction of the principal coordinate analysis (PCOA) graph was performed using the QIIME2 package. A linear discriminant analysis (LEFse) effect size measure (Segata et al., 2011) was used to compare the relative percentages of individual bacterial taxonomic units in the microbiomes of the matched groups.

Statistical processing of the study results was performed using the STATISTICA.10 program package (Statsoft, USA). Quantitative parameters were evaluated by calculating mean values (M). The Mann–Whitney rank U-test was used to assess the reliability of differences in the relative percentages of individual bacterial taxa in the samples. Differences were considered reliable at p < 0.05. To eliminate the effect of multiple comparisons, the False Discovery Rate (FDR) correction was used to assess the significance of differences. Multiple regression analysis was used to assess the relationships between the content of individual bacteria in the sputum of patients with the presence of comorbidities, smoking, alcohol consumption, place of residence, and occupational harmful factors.

## Results

Sequencing of the V3-V4 region of the 16S rRNA gene in sputum identified a total of nine bacterial types with a relative frequency above 0.1 %. The predominant bacterial types in the microbiomes of LUSC patients, AD patients and controls were Bacillota and Bacteroidota, which together accounted for about 70 % of the total microbiota. Overall, the relative percentages as well as the ratio of dominant bacterial types in sputum appeared close to the parameters previously described for the sputum microbiome in LC patients (Hosgood et al., 2014; Huang et al., 2019).

**Fig. 1.**
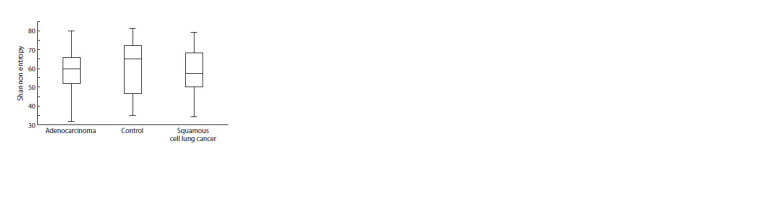
Shannon diversity index of microbiomes of patients with adenocarcinoma, squamous cell lung cancer and control donors.

**Fig. 2.**
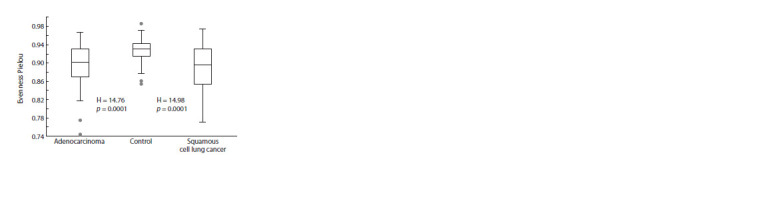
Pielou index of microbiomes of patients with adenocarcinoma, squamous cell lung cancer and control donors

The Shannon index was used to assess alpha diversity. The results of the analysis showed that there were no differences between the matched samples of patients and healthy donors (Fig. 1). However, a significant reduction in alpha diversity according to the Pielou index (evenness) was found in the sputum of patients with AD and with LUSC compared to controls (Kruskal–Wallis test; p = 0.0001). There were no significant differences in uniformity (Pielou index) between the different histologic types of LC (Fig. 2).

**Fig. 3.**
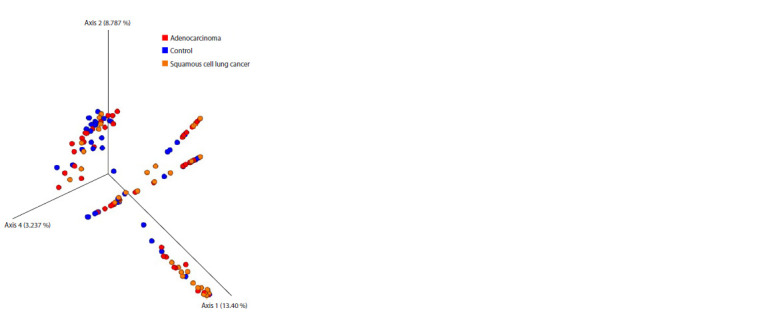
Three-dimensional diagram constructed by principal component analysis showing the phylogenetic diversity of prokaryotic communities in the sputum of patients with adenocarcinoma, squamous cell lung cancer and control donors

Differences in bacterial community structure (beta diversity) in sputum samples from AD patients, LUSC patients and healthy individuals were evaluated with the PERMANOVA (Adonis) test using a Bray–Curtis difference matrix (Fig. 3). The analysis showed that there were differences in beta diversity only between LUSC and control communities (pseudo- F = 3.89; p = 0.007).

**Fig. 4.**
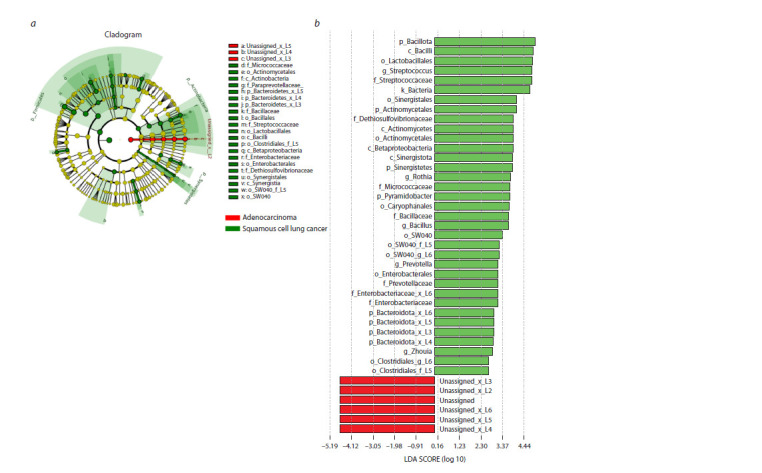
Different representation of bacterial taxa in sputum samples of patients with squamous cell carcinoma and adenocarcinoma of the lung. Here and in Fig. 5, 6: a – cladogram giving an idea of the proximity of the differing taxonomic groups; b – graph representing the results of LEFse analysis. LDA – linear discriminant analysis.

**Fig. 5.**
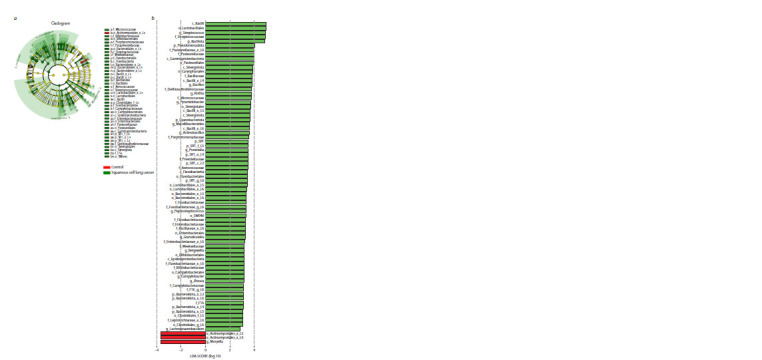
Different representation of bacterial taxa in sputum samples of patients with squamous cell cancer and healthy donors

**Fig. 6.**
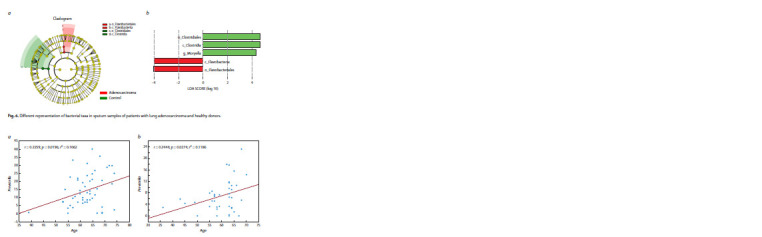
Different representation of bacterial taxa in sputum samples of patients with lung adenocarcinoma and healthy donors.

Differences in bacterial taxonomic composition between the study samples were examined using linear discriminant analysis (LEFse), which estimates the effect size of the representation of different bacteria. The LEFse method revealed a significant increase in the representation of selected bacterial taxa in the sputum of patients with LUSC compared with AD. This applies in particular to the type Bacillota, the class Bacilli and the genus Streptococcus (Fig. 4). Comparison of the taxonomic composition of LUSC patients and healthy donors showed an increase in the content of representatives of Bacillota and Pseudomonadota types, Bacilli class, genera Streptococcus, Rothia, Bacillus, Macellibacteroides, etc. in the sputum of patients (Fig. 5). There were significantly fewer bacterial taxa for which LEFse analysis revealed differences between healthy donors and AD patients (Fig. 6). Specifically, the sputum of healthy individuals showed an increase in representatives of the order Clostridiales, the class Clostridia and the genus Moryella, whereas in patients with AD the representation of the order Flavobacteriales and the class Flavobacteriia was increased.

Multiple regression analysis (MRA) was used to assess possible relationships between the content of individual bacteria in the sputum of LUSC patients with a range of other factors potentially affecting the composition of the microbiota. In addition to the bacterial genera (Streptococcus, Rothia, Bacillus, Macellibacteroides) significant for LUSC, MRA models included sex, patient age, comorbidities, smoking, alcohol consumption, place of residence, and presence of occupational hazards (see Table 1). As a result, it was found that among the confounders studied, only the presence of cardiovascular disease (ischemia, hypertension, etc.), chronic bronchitis, and/or chronic obstructive pulmonary disease was associated with LUSC.

**Fig. 7.**
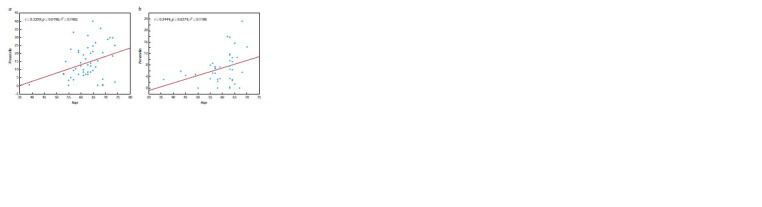
Content of Prevotella representatives in the sputum of patients with lung adenocarcinoma (a) and squamous cell lung cancer (b) as a function of age.

The effect of age and smoking status on microbiota composition in patients and controls was studied separately. Correlation analysis (Spearman) revealed a significant increase with age in the content of Prevotella species in the sputum of patients with AD ( p = 0.0196) and in patients with PRL ( p = 0.0274) (Fig. 7). At the same time, a positive correlation of age with the content of representatives of the genera Atopobium ( p = 0.03) and Leptotrichia ( p = 0.03) was observed in patients with AD. In the control samples, an increase with age in the content of bacteria from the genera Porphyromonas ( p = 0.01) and Veillonella ( p = 0.045) and, at the same time, a decrease in the content of representatives of the genera Lachnoanaerobaculum ( p = 0.02), Stomatobaculum ( p = 0.006) and Oribacterium ( p = 0.02) were found.

Smoking status had no effect on sputum microbiome composition in patients with AD and LUSC. For the control sample, there was an increase in Streptococcus in the sputum of smoking donors compared to nonsmoking donors (20.87 vs. 15.16 %; p = 0.0007), and a significant decrease in Neisseria in the sputum of smokers (2.75 vs. 5.68 %; p = 0.001).

A question of separate interest is the possible influence of the stage of the tumor process on the composition of bacteria in sputum. The results summarized in Table 2 show that the percentage of bacterial taxa differs significantly between patients with NSCL in stages I–II as compared to stages III–IV of the disease. From the analysis of this data, it can be concluded that there is an increase in bacteria belonging to four genera in the sputum of patients in advanced stages of tumorigenesis.

**Table 2.**
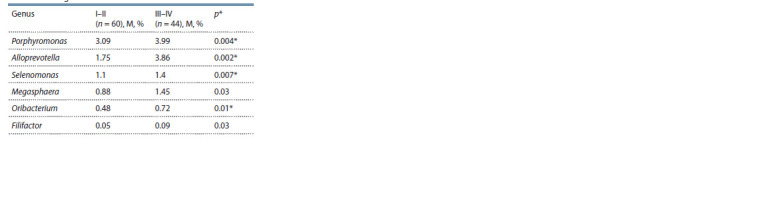
Average percentage of bacterial taxa in the sputum microbiome of patients with non-small cell lung cancer at different stages of the disease Here and in Table 3: the p-value is less than the FDR-corrected p-value.

Primary tumor localization in LC may be another factor potentially influencing the composition of the bacterial microbiota of the respiratory tract. Therefore, we compared the mean percentage of bacterial genera in the sputum of patients with central NSCLC and peripheral NSCLC (Table 3).

**Table 3.**
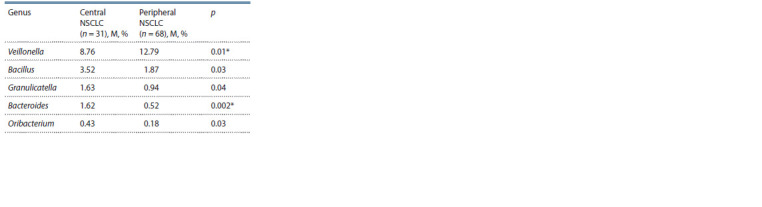
Average percentage of bacterial taxa in the sputum microbiome of patients with non-small cell lung cancer with different tumor localization

As follows from these data, the central localization of the tumor is accompanied by an increase in representatives of the genus Bacteroides. At the same time, an increase in bacteria of the genus Viellonella was observed in patients with peripheral NSCLC as compared to a central tumor localization (12.79 vs. 7.99 %; p = 0.01).

## Discussion

Differences in the taxonomic composition of the bacterial microbiome of the human respiratory tract have already been recognized as an important pathogenetic factor in lung cancer (Maddi et al., 2019; Yagi et al., 2021), but to date, the importance of the microflora in patients with different histological types of NSCLC remains an open question. Here, we compared the taxonomic composition of the microbiome in sputum samples from patients with the two most common forms of NSCLC: adenocarcinoma and squamous cell lung cancer.

According to previous studies, the respiratory microbiota of LC patients tends to have lower alpha diversity compared to healthy individuals, while beta diversity is not significantly different (Lee et al., 2016; Liu N.N. et al., 2020). The same trend has been observed for the microbiomes of cancer-affected and non-cancerous lung tissues (Kim et al., 2022).

Evidence of similarities or differences between airway and lung tissue community diversity parameters of patients with different histologic types of LC to date is scarce and these results are inconsistent. For example, alpha diversity of the microbiome was found to be higher in the sputum of AD patients compared to LUSC and a significant difference in beta diversity was also found between these groups, but the samples compared were too small (6 and 7 cases, respectively) (Ran et al., 2020). Another study showed that the bronchoalveolar lavage (BAL) microbiota was more diverse in LUSC than in AD (Gomes et al., 2019). No differences in alpha diversity as well as beta diversity of microbiomes from sputum and BAL samples were found between samples of patients with AD and LUSC (Huang et al., 2019). The microbiome of tumor tissues of patients with AD did not differ in alpha diversity from LUSC, although a significant increase in the content of Gram-positive bacteria was recorded in the adenocarcinoma group (Kovaleva et al., 2020).

Our study showed that the values of the Shannon index, which reflects the species richness of the microbiota, are close in the matched cohorts of patients and control donors. A significant decrease in both patient cohorts compared to controls was observed for the evenness index, which is based on measuring the relative abundance of different species in a community and is one of the metrics characterizing alpha diversity. Bacterial community structures (beta diversity) between AD and LUSC were also similar, but according to the Bray– Curtis matrix, differences were present between the bacterial communities of LUSC patients and healthy subjects, but not between AD and controls (see Fig. 3). Thus, our study showed that the α-diversity and β-diversity of bacterial communities of sputum from patients with different histologic types are similar. Nevertheless, it is worth noting that the microbiome of LUSC patients differs significantly from that of healthy individuals.

To answer the question of differences between sputum microbiome compositions in cohorts of patients with different histologic types of LC, we used the LEFse method, which is the most commonly used method in microbiome studies. LEFse analysis allowed identification of differences between the compared patient samples (see Fig. 4). The sputum of LUSC patients had a significant enrichment of Bacillota (genus Streptococcus and Bacillus) and Actinomycetota (genus Rothia) when compared with samples from AD patients. Comparison of respiratory microbiome composition in groups of patients with LUSC and healthy subjects also revealed a number of significant differences. According to the results of LEFse analysis (see Fig. 5), the content of representatives of the phylum Bacillota and Pseudomonadota; genera Streptococcus, Bacillus, Rothia, Macellibacteroides, Prevotella, Actinobacillus and Peptostreptococcus was increased in the sputum of patients compared to controls. In healthy study participants, an increase in the representation of the Actinomycetales and the genus Moryella was observed.

Several previous studies have shown that the composition of the bacterial microbiota in the respiratory tract of LC patients may be histologically dependent. For example, Q. Leng and colleagues (Leng et al., 2021) used digital droplet PCR to analyze 25 genera of bacteria commonly associated with NSCLC in the sputum of 17 NSCLC patients and 10 healthy subjects. A significant increase in the content of representatives of the genera Acidovorax, Streptococcus, H. pylori and Veillonella was detected in the sputum of LUSC patients, whereas an increased abundance of Capnocytophaga was found in the sputum of AD patients. These same sputum bacterial biomarkers were then confirmed in another cohort consisting of 69 NSCLC cases and 79 control donors. In another study, the relationship between saliva microflora and lung cancer was examined. DNA samples from 20 LC patients (10 LUSC and 10 AD) and control subjects (n = 10) were sequenced (Yan et al., 2015). At the level of bacterial genera, Capnocytophaga, Selenomonas, and Veilonella were elevated in both AD and squamous cell cancer, and Neisseria was reduced in both AD and LUSC.

In our study, patients with LUSC had a significant increase in members of the genera Streptococcus, Bacillus and Rothia compared to AD. There was an increase in Capnocytophaga (1.46 vs. 1.08 %) in the sputum of AD patients compared to LUSC, as in a previous study (Leng et al., 2021), but these differences were not significant. Thus, it can be stated on the one hand that the two main histologic forms of LC have distinct respiratory microbiomes, however, there is no uniform set of bacterial taxa marking these differences. Perhaps, this fact reflects the initially different composition of bacteria inhabiting the respiratory tract of patients with NSCLC living in different regions of the world, i. e. it is a consequence of environmental factors (Costello et al., 2012).

An important finding of this study is the significant difference in the content of bacterial taxa in the sputum microbiome of patients with different histologic forms of LC compared to healthy subjects. While for LUSC there is a significant enrichment of Streptococcus, Bacillus, Rothia, Macellibacteroides, Prevotella, Actinobacillus and Peptostreptococcus genera in sputum (see Fig. 5), no significant differences in bacterial composition were found in the sample of patients with adenocarcinoma compared to controls (see Fig. 6). This fact means that the search for metagenomic biomarkers associated with LC can be correct only after separate analysis of microbiota composition depending on the histological classification of the tumor.

The sample size used in our study allowed us to examine, in addition to the histological type of tumor, other individual factors (age, smoking status, stage of malignant process, tumor localization) potentially capable of influencing the composition of the microbiota in NSCLC. Of interest is the age-correlated increase in the content of representatives of the genus Prevotella, which was registered in both samples of patients (see Fig. 7). This is in disagreement with the results of a study of BAL samples from NSCLC patients, where a subgroup of patients older than 60 years recorded a decrease in Prevotella (P. oryzae) compared to younger patients (Zheng et al., 2021).

Comparison of the composition of the sputum microbiome in smoking and nonsmoking patients with PRL and ACL showed no differences in bacterial composition. However, the control group showed an increase in Streptococcus as well as a marked decrease in Neisseria in the sputum of smokers compared to nonsmokers, which is consistent with previously published results (Huang, Shi, 2019; Ying et al., 2022). Noteworthy is the fact that smokers also show increased Streptococcus representation and decreased Neisseria representation in the upper gastrointestinal tract compared to non-smoking donors (Shanahan et al., 2018). According to recent findings (Haldar et al., 2020), the effect of smoking on sputum microbiota remains unclear and requires further investigation.

The evaluation of the possible influence of NSCLC stage on the structure of sputum microbiome has shown that in the sputum of patients at advanced stages of tumor progression there is an increase in the content of bacteria belonging to the genera Porphyromonas, Alloprevotella, Selenomonas, Megasphaera, Oribacterium and Filifactor. NSCLC patients with central lung cancer had increased sputum levels of bacteria from the genus Bacteroides. At the same time, an increase in Veillonella content was noted in patients with peripheral lung cancer compared to central tumor localization. These results should be considered as preliminary, as the analysis was performed for the total sample of patients without taking into account the histologic type of NSCLC

## Conclusion

In this study, a comparative analysis of the taxonomic composition of the bacterial microbiome of sputum from patients with two major histologic types of NSCLC and healthy sputum donors was performed based on the sequence of the 16S rRNA coding gene region identified using massively parallel sequencing technology. Significant differences in the content of representatives of a number of bacterial genera in the sputum of patients with LUSC and AD were revealed. In particular, the presence of Streptococcus, Bacillus, Rothia and other genera was elevated in the sputum of LUSC patients compared to healthy subjects.

The present findings require confirmation in independent large-scale studies to further understand the role of the sputum microbiota in the development of NSCLC. In addition, the search for bacterial “signatures” associated with lung cancer risk requires whole-genome sequencing to obtain an accurate assessment of taxonomic composition at the species level.

## Conflict of interest

The authors declare no conflict of interest.
